# Effects of Single-Cell Protein Derived from *Ralstonia eutropha* on Growth Performance, Meat Quality, Antioxidant Capacity, and Intestinal Health in Broilers

**DOI:** 10.3390/ani16172769

**Published:** 2026-09-03

**Authors:** Xiaoyao Duan, Minglu Guo, Zhiyong Rao, Wei Zhang, Zhixiang Wang, Yongpeng Guo

**Affiliations:** College of Animal Science and Technology, Henan Agricultural University, Zhengzhou 450046, China; duanxy017@163.com (X.D.); 17734734361@163.com (M.G.); rzyhau@163.com (Z.R.); weizhang@henau.edu.cn (W.Z.)

**Keywords:** *Ralstonia eutropha* H16, single-cell protein, broilers, growth performance, antioxidant capacity, intestinal health

## Abstract

This study aims to evaluate the feasibility of substituting soybean meal with single-cell protein derived from *Ralstonia eutropha* H16 (SCP-H16) in broiler diets, with a specific emphasis on its impact on growth performance, meat quality, biochemical parameters, antioxidant capacity, and intestinal health. A total of 540 one-day-old Arbor Acres (AA) male broiler chicks were randomly allocated to five treatment groups, each comprising six replicates of 18 chicks. The control group (CON) was provided with a corn-soybean meal basal diet, whereas the experimental groups received the basal diet supplemented with 2%, 4%, 6%, and 8% SCP-H16, respectively, replacing soybean meal on an isonitrogenous basis. Specifically, dietary inclusion of 2%, 4%, 6%, and 8% SCP-H16 replaced approximately 9%, 18%, 27%, and 36% of the soybean meal in the starter diet and 10%, 21%, 31%, and 41% in the grower diet, respectively. The feeding period lasted 42 days. The findings demonstrated that dietary supplementation with SCP-H16 significantly reduced the average daily feed intake and feed conversion ratio of broilers (*p* < 0.05), without adversely affecting the final body weight and breast meat quality at 42 d. Compared to the CON group, the groups receiving 2% and 4% SCP-H16 exhibited significantly lower serum level of total cholesterol (T-CHO) (*p* < 0.05). Moreover, the serum activity of total superoxide dismutase (T-SOD) was significantly enhanced in the 2%, 4%, and 6% SCP-H16 groups, and the total antioxidant capacity (T-AOC) was significantly increased in the 2% and 4% SCP-H16 groups (*p* < 0.05), relative to the CON group. In the liver, glutathione peroxidase (GSH-Px) activity was significantly elevated in the 2% SCP-H16 group compared to the CON group (*p* < 0.05). Additionally, serum levels of IgG and IgM were significantly elevated in the 2%, 4%, and 6% SCP-H16 groups, while serum IgA level was increased across all SCP-H16-containing groups (*p* < 0.05). In the liver, both IgG and IgA levels were significantly elevated across all groups receiving SCP-H16 supplementation (*p* < 0.05). In the duodenum, the 2% SCP-H16 group exhibited a significantly greater villus height compared to the CON group (*p* < 0.05). Furthermore, in both the duodenum and ileum, the groups receiving 2% and 4% SCP-H16 demonstrated a significant reduction in crypt depth and an increased villus height-to-crypt depth ratio relative to the CON group (*p* < 0.05). The 2% SCP-H16 group also showed significantly elevated mRNA expression levels of Occludin and zonula occludens-1 (*ZO-1*) in the duodenum compared to the CON group (*p* < 0.05). In the ileum, the 2% and 4% SCP-H16 groups exhibited significantly increased Occludin mRNA expression relative to the CON group (*p* < 0.05), with the 2% SCP-H16 group also showing a significant increase in zonula occludens-1 (*ZO-1*) mRNA expression (*p* < 0.05). In conclusion, dietary supplementation with 2% to 4% SCP-H16 is associated with enhanced antioxidant capacity, elevated humoral immunity, and improved intestinal morphology and barrier-related gene expression, suggesting that SCP-H16 can serve as a promising alternative protein source to soybean meal.

## Simple Summary

Given the current scarcity of protein resources within the animal husbandry sector, it is essential to identify alternatives that are cost-effective, nutritionally adequate, and sustainable to address the shortage of soybean meal. This study aimed to investigate the effects of replacing soybean meal with single-cell protein derived from *Ralstonia eutropha* H16 (SCP-H16) on the growth performance, meat quality, antioxidant capacity, and intestinal health of broilers. The results demonstrated that SCP-H16 did not adversely affect growth performance or meat quality; instead, it enhanced antioxidant capacity and immune status and promoted intestinal health. These findings highlight the potential of SCP-H16 as an innovative protein source to substitute for soybean meal.

## 1. Introduction

The global poultry industry has experienced significant expansion to satisfy the increasing demand for high-quality animal protein, with broiler production playing a pivotal role in this growth [1]. Within the economic structure of modern broiler farming, feed costs constitute the most substantial operational expenditure, typically comprising approximately 70% of the total production budget. Among feed formulations, protein sources are a primary cost determinant. Soybean meal has traditionally been the benchmark in monogastric nutrition due to its favorable amino acid profile and high protein digestibility, making it the predominant protein ingredient in broiler diets globally [2,3]. However, recent fluctuations in global grain production and rising energy costs have led to considerable volatility in soybean meal prices, thereby increasing feed expenses and posing a threat to the sustainable development of the poultry sector [4]. Therefore, the development of cost-effective, sustainable, and nutritionally adequate alternatives to soybean meal has become an urgent priority in animal nutrition research.

In addressing these challenges, recent advancements in biotechnology have positioned microbial biomass, commonly referred to as single-cell protein (SCP), as a crucial resource for ensuring future food and feed security [5]. Unlike traditional protein production systems that rely on plants and animals, SCP offers several significant advantages, including independence from climatic and seasonal variations, a rapid production cycle, minimal environmental impact, high productivity, and the ability to efficiently convert diverse substrates into protein [6]. Moreover, SCP production can be seamlessly integrated into circular bioeconomy models by utilizing agro-industrial waste or even greenhouse gases as fermentation feedstocks [7]. Due to these advantages, SCP derived from bacteria, yeast, algae, and fungi is increasingly being investigated and utilized as a protein source in both human food ingredients and animal feed formulations.

Among the various microbial candidates, *Ralstonia eutropha*, also referred to as *Cupriavidus necator*, has emerged as a particularly promising bacterium for SCP production. This Gram-negative, facultative chemolithoautotroph is distinguished by its robust growth characteristics and its capacity to accumulate high levels of cellular biomass [8,9]. Notably, the crude protein content of *R. eutropha* can constitute between 69% and 83% of its cellular dry weight, being comparable to or higher than many yeast and fungal SCPs [10]. The strain *R. eutropha* H16 is especially notable as an expression host due to its well-characterized genetics and metabolic versatility [11]. Crucially, toxicological assessments have demonstrated that SCP derived from *R. eutropha* H16 (SCP-H16) exhibits no cytotoxic or genotoxic effects in murine models, highlighting its potential as a safe, nutritious, and sustainable protein source for diverse applications [12]. In addition to its nutritional attributes, *R. eutropha* H16 biomass comprises bioactive peptides, nucleotides, and peptidoglycans, which may have the potential to modulate the composition of gut microbiota, enhance intestinal barrier function, and confer antioxidant benefits by improving nutrient absorption and reducing oxidative stress. Despite these compelling attributes, the practical application of SCP-H16 in monogastric nutrition remains largely unexplored. To date, no systematic evaluation of SCP-H16 as a soybean meal substitute in broiler diets has been reported, and its effects on growth performance, meat quality, antioxidant capacity, immune function, and intestinal health in fast-growing chickens are entirely unknown. Therefore, the objective of this study was to systematically evaluate the feasibility of replacing dietary soybean meal with SCP-H16 in broiler diets and to investigate its impact on these parameters. The findings aim to provide a scientific foundation for the utilization of this novel bacterial protein in sustainable poultry production systems.

## 2. Materials and Methods

### 2.1. Experimental Design and Bird Management

Single-cell protein derived from *Ralstonia eutropha* H16 (SCP-H16) was supplied by the Tianjin Institute of Industrial Biotechnology, Chinese Academy of Sciences (Tianjing, China). A total of 540 one-day-old Arbor Acres (AA) male broiler chicks were randomly allocated into five treatment groups, each comprising six replicates of 18 chicks (*n* = 6 pens/treatment, 18 birds/pen). The control group was provided with a corn-soybean meal basal diet, while the experimental groups received the basal diet supplemented with 2%, 4%, 6%, and 8% SCP-H16, respectively, as a replacement for soybean meal on an isonitrogenous basis. The experimental duration was 42 days. Birds were reared in an environmentally controlled broiler house. During the first week, the temperature within the chicken house was maintained at 35–36 °C, subsequently decreasing by 2 °C per week until reaching 25 °C. The relative humidity within the chicken house was maintained at approximately 60%, with continuous 24 h illumination achieved through natural light supplemented by auxiliary LED lighting (warm white, 3000 K, approximately 20 lux intensity) during nighttime. Feed was provided as a ground mash and offered ad libitum. Broilers had 24 h access to clean drinking water at room temperature. All broilers were administered a live attenuated combined NDV-IBV vaccine (La Sota + H120) via drinking water on days 7, 14, and 28, in accordance with the standard immunization schedule. The basal diet, primarily composed of corn and soybean meal, was formulated in compliance with the Chinese Feeding Standard for Broilers (GB/T5916-2020). The composition and nutritional content of the diets for each experimental group are detailed in Table 1 and Table 2, while the nutritional composition of SCP-H16 is presented in Table S1.

### 2.2. Growth Performance

Body weight (BW) of broilers was measured by pen on the mornings of days 1, 21, and 42 of age. Concurrently, data on feed consumption and survival rates per pen were recorded. These measurements facilitated the subsequent calculation of average daily gain (ADG), average daily feed intake (ADFI), and feed conversion ratio (FCR).

### 2.3. Sample Collection

On the 42nd day of the feeding experiment, one chicken with a body weight closest to the pen mean was selected from each replicate (6 birds per treatment, total 30 birds) by an investigator who was blinded to the treatment allocation. Blood samples were obtained from the wing vein and subjected to centrifugation at 3000× *g* for 15 min at 4 °C to separate the serum, which was subsequently frozen at −20 °C for future analysis. Post blood collection, the broilers were humanely euthanized via cervical dislocation. The abdominal cavity was subsequently opened, and liver tissue was collected and preserved in cryovials. Additionally, 3 cm segments from the anterior sections of the duodenum, jejunum, and ileum were excised and placed in 4% paraformaldehyde fixative, while the remaining duodenal, jejunal, and ileal tissues, along with the liver tissue, were stored at −80 °C for subsequent analysis.

### 2.4. Meat Quality

Following exsanguination and evisceration, standardized tissue samples were collected from the left *pectoralis major* muscle of each broiler carcass. Muscle color parameters, including lightness (L*), redness (a*), and yellowness (b*), were measured at 45 min postmortem using a chroma meter (CR-400, Konica Minolta Holdings, Inc., Tokyo, Japan). Three random locations on each sample were measured, and the average value was recorded. Measurements were repeated after 24 h of storage at 4 °C. Additionally, muscle pH was determined at both 45 min and 24 h postmortem using a portable pH meter (Testo 205, Testo AG, Lenzkirch, Germany), which was inserted directly into the muscle to a depth of approximately 1 cm. Three readings were taken per sample, and the average value was calculated.

Water-holding capacity was evaluated via drip loss and cooking loss. Drip loss was quantified by initially weighing approximately 3 g of breast muscle tissue, which was then placed in a drip loss tube and stored at 4 °C for 24 h prior to reweighing. Cooking loss was determined by sealing samples in polyethylene bags and heating them in 80 °C water bath until the internal temperature reached 70 °C. Samples were then cooled to room temperature, dried, and reweighed. Drip loss and cooking loss were calculated as the percentage of weight lost relative to the initial weight.

### 2.5. Serum Biochemical Indices

The serum concentrations of alanine aminotransferase (ALT), aspartate aminotransferase (AST), alkaline phosphatase (ALP), total protein (TP), albumin (ALB), globulin (GLO), total bilirubin (TBIL), creatinine (CREA), uric acid (UA), glucose (GLU), and total cholesterol (T-CHO) were determined using a fully automated biochemical analyzer (Mindray BS-330E, Shenzhen Mindray Bio-Medical Electronics Co., Ltd., Shenzhen, China).

### 2.6. Antioxidant Capacity of Serum and Liver

The activities of glutathione peroxidase (GSH-Px; Catalog No. A005-1-2) and total superoxide dismutase (T-SOD; Catalog No. A001-1-2), as well as the concentration of malondialdehyde (MDA; Catalog No. A003-1-2) and total antioxidant capacity (T-AOC; Catalog No. A015-3-1) in the serum and liver of broilers were determined using commercial kits (Nanjing Jiancheng Bioengineering Institute, Nanjing, China) in accordance with the manufacturer’s instructions.

### 2.7. Immunoglobulin Levels in Serum and Liver

The levels of IgA, IgG, and IgM in serum and liver samples from each treatment group were determined in accordance with the instructions of the Chicken Immunoglobulin ELISA Kit (Xiamen Lunchangshuo Biotechnology Co., Ltd., Xiamen, China).

### 2.8. Intestinal Morphology

Samples from the duodenum, jejunum, and ileum, which had been previously fixed in a 4% paraformaldehyde solution, were retrieved and subjected to a dehydration process using a graded ethanol series. The samples were then cleared in a xylene/absolute ethanol mixture (1:1, *v*/*v*) for 2 to 3 min before being embedded in paraffin. The paraffin-embedded tissue blocks were sectioned at a thickness of 3 to 5 μm and stained with hematoxylin and eosin (H&E). Subsequently, the slides were scanned using a KF-FL-400 digital slide scanner (Ningbo Konfoong Bioinformation Tech Co., Ltd., Ningbo, China) and examined under a microscope (Nikon E200, Nikon Corporation, Tokyo, Japan). For each intestinal segment, at least 10 well-oriented, intact villi and their corresponding crypts were measured per section by an evaluator who was blinded to treatment groups. Measurements of villus height (VH), crypt depth (CD), and the ratio of VH to CD (VH/CD) in the duodenum, jejunum, and ileum were conducted using ImageJ software J2.

### 2.9. Quantitative Real-Time PCR Analysis

Total RNA was extracted from duodenal, jejunal, and ileal tissues using the Trizol extraction method. The RNA concentration and purity were measured using a UV-Vis spectrophotometer(Shanghai Metash Instruments Co., Ltd., Shanghai, China). RNA quality was confirmed by A260/A280 ratios and agarose gel electrophoresis. Subsequently, the extracted RNA was reverse-transcribed into cDNA following the protocol provided by the manufacturer of the PrimeScript™ FAST RT Reagent Kit with gDNA Eraser. Quantitative real-time PCR was conducted on a CFX96™ Real-Time System (Bio-Rad Laboratories, Inc., Hercules, CA, USA), utilizing the 2 × ChamQ Universal SYBR qPCR Master Mix in accordance with the manufacturer’s guidelines. Primer amplification efficiencies, determined by standard curve analysis, ranged from 95% to 105%. GAPDH was selected as the reference gene based on its stable expression across treatments in preliminary tests and its established use as a housekeeping gene in broiler intestinal studies. The relative mRNA expression levels of target genes were calculated using the 2^−ΔΔCt^ method with GAPDH as the internal reference gene. The primer sequences are listed in Table S2.

### 2.10. Statistical Analysis

Statistical analyses of the experimental data were performed using SPSS software (version 27.0; SPSS Inc., Chicago, IL, USA). For growth performance, the pen served as the experimental unit (*n* = 6). For meat quality, serum and liver biochemistry, intestinal morphology, and gene expression analyses, one bird was randomly selected from each pen, and thus the experimental unit for these analyses was also the pen (*n* = 6 pens/treatment), with individual birds serving as observational units representing their respective pens. Prior to the analysis of variance, Shapiro–Wilk’s test was performed to check the normality, and Levene’s test was conducted for homogeneity of variance. One-way analysis of variance (ANOVA) was first conducted, followed by Tukey’s test for multiple comparisons. Given the exploratory nature of this study, no additional formal corrections for multiple comparisons were applied beyond Tukey’s test for pairwise comparisons. All data are expressed as mean ± pooled standard error of the mean (SEM) and statistical significance was defined as *p* < 0.05.

## 3. Results

### 3.1. Growth Performance

As shown in Table 3, during days 1 to 21 of the experiment, all SCP-H16 groups showed a statistically significant reduction in final body weight and average daily gain compared to the control group (*p* < 0.05). Additionally, the average daily feed intake was significantly reduced in the 6% and 8% SCP-H16 groups (*p* < 0.05). During days 22 to 42, all SCP-H16 groups exhibited a significant decrease in feed conversion ratio relative to the control group (*p* < 0.05), with a notable reduction in average daily feed intake observed in the 2%, 6%, and 8% SCP-H16 groups (*p* < 0.05). Over the entire experimental period, both the average daily feed intake and feed conversion ratio in all SCP-H16 groups were significantly lower than those in the control group (*p* < 0.05). However, there was no significant difference in body weight at 42 days of age among all groups (*p* > 0.05). In summary, while SCP-H16 reduced the average daily feed intake in broilers, it enhanced feed conversion efficiency and did not adversely affect broiler growth. The decoupling of reduced feed intake from growth maintenance constitutes a desirable outcome in commercial broiler production.

### 3.2. Meat Quality

Table 4 presents the findings related to breast muscle quality. The administration of SCP-H16 did not exhibit a statistically significant impact on the pH, lightness (L*), redness (a*), and yellowness (b*) parameters, nor on the drip loss and cooking loss of the breast muscle (*p* > 0.05).

### 3.3. Serum Biochemical Indices

The results of the analysis of serum biochemical parameters in broiler chickens are presented in Table 5. In comparison to the control group, the groups receiving 2% and 4% SCP-H16 demonstrated significantly reduced levels of T-CHO (*p* < 0.05). However, no significant differences were detected among the groups concerning serum levels of ALT, AST, ALP, TP, ALB, GLO, TBIL, CREA, UA, and GLU (*p* > 0.05).

### 3.4. Antioxidant Capacity of Serum and Liver

The impact of SCP-H16 on the antioxidant capacity of serum and liver in broilers is presented in Table 6. In the serum, there was a statistically significant increase in T-SOD activity in the 2%, 4%, and 6% SCP-H16 groups, while the T-AOC levels were significantly higher in the 2% and 4% SCP-H16 groups compared to the control group (*p* < 0.05). In the liver, GSH-Px activity was significantly enhanced in the 2% SCP-H16 group relative to the control group (*p* < 0.05). No significant differences were observed in serum MDA and GSH-Px levels, or in liver T-SOD, T-AOC, and MDA levels among the groups (*p* > 0.05).

### 3.5. Immunoglobulin Levels in Serum and Liver

Table 7 presents the impact of SCP-H16 on immunoglobulin concentrations in the serum and liver of broilers. In the serum, the levels of IgG and IgM were significantly higher in the 2%, 4%, and 6% SCP-H16 groups compared to the control group (*p* < 0.05), whereas IgA levels were significantly elevated across all SCP-H16 groups (*p* < 0.05). In the liver, both IgG and IgA concentrations were significantly increased in all groups receiving SCP-H16 compared to the control group (*p* < 0.05). No significant difference was observed in liver IgM levels among the groups (*p* > 0.05).

### 3.6. Intestinal Morphology

The findings from the analysis of intestinal morphology are detailed in Table 8. In the duodenum, the group receiving 2% SCP-H16 demonstrated a significantly greater villus height compared to the control group (*p* < 0.05). Furthermore, in both the duodenum and ileum, the groups administered 2% and 4% SCP-H16 exhibited a significant reduction in crypt depth and an increased villus height-to-crypt depth ratio relative to the control group (*p* < 0.05). No significant differences were observed in jejunal morphology (villus height, crypt depth, and VH/CD ratio) among all groups (*p* > 0.05). Additionally, duodenal villus height in the 6% and 8% SCP-H16 groups, as well as ileal villus height across all SCP-H16 groups, did not differ significantly from the control (*p* > 0.05).

### 3.7. Expression of Tight Junction Protein-Related Genes

As illustrated in Table 9, the expression levels of Occludin and ZO-1 in the duodenum were significantly elevated in the 2% SCP-H16 group compared to the control group (*p* < 0.05). In the jejunum, the expression level of ZO-1 was significantly higher in the 2% SCP-H16 group than in the 8% SCP-H16 group (*p* < 0.05), while no significant differences were observed in jejunal Occludin expression among the groups (*p* > 0.05). Furthermore, in the ileum, the 2% and 4% SCP-H16 groups exhibited significantly increased Occludin expression, and the 2% SCP-H16 group also showed significantly increased ZO-1 expression relative to the control group (*p* < 0.05). No significant differences in *Claudin-1* gene expression were detected among the groups across the duodenum, jejunum, and ileum (*p* > 0.05).

## 4. Discussion

Growth performance is a crucial metric for assessing the productivity and economic feasibility of broiler production. Existing research suggests that substituting soybean meal with enzymatically hydrolyzed cottonseed protein in broiler diets does not significantly impact body weight, daily weight gain, or feed conversion ratio [14]. Dayan et al. [15] found that incorporating 2% to 6% torula yeast-derived SCP as a partial replacement for soybean meal in broiler feed did not significantly affect growth performance throughout the trial period. Moreover, Shan et al. [16] demonstrated that replacing soybean meal with *Clostridium autoethanogenum* protein does not negatively impact the growth performance of broiler chickens while improving feed conversion efficiency. In the present study, dietary SCP-H16 substitution did not compromise final body weight at day 42 and significantly improved feed conversion efficiency across all inclusion levels. Notably, broilers exhibited reduced ADG during the starter phase (1–21 d) alongside decreased ADFI throughout the trial. Early-growth depression may compromise flock uniformity and early immune development. This transient early-phase suppression likely reflects an adaptive period to the distinct peptide structures and amino acid profile of bacterial protein, consistent with observations reported for *Clostridium autoethanogenum* SCP [16] and torula yeast SCP [15]. However, this explanation remains speculative, as nutrient digestibility, intestinal microbiota composition, and amino acid utilization were not evaluated in the present study. Further research incorporating these measurements is needed to confirm this hypothesis. Notably, the experimental diets were formulated based on total amino acid content rather than digestible amino acid values. Amino acid digestibility varies widely among protein sources, especially when comparing plant-based soybean meal with bacterial single-cell protein. Furthermore, the inclusion of H16 SCP had no detrimental effects on breast meat quality attributes, including pH, color, cooking loss, and drip loss. These findings indicate that SCP-H16 serves as a viable alternative to soybean meal, exhibiting no detrimental impact on broiler growth performance and breast meat quality.

Serum biochemical parameters provide critical insights into the metabolic status and health of animals. Prior research has demonstrated that dietary supplementation with SCP derived from *Nannochloropsis oculata* effectively reduces serum cholesterol levels in laying hens [17]. Consistent with these findings, the present study indicates that dietary supplementation with 2% and 4% SCP-H16, as a replacement for soybean meal, significantly decreases serum total cholesterol levels in broiler chickens. The antioxidant capacity is intricately linked to the health status of poultry, with the enzymatic activities of T-SOD and GSH-Px, in conjunction with the T-AOC level, serving as critical biomarkers for evaluating antioxidant capacity in animals. Previous research has shown that dietary supplementation with an appropriate amount of SCP derived from *Clostridium autoethanogenum* can enhance serum GSH-Px and SOD activities, as well as T-AOC, thereby improving the antioxidative capacity of broilers [18]. The findings of the present study corroborate previous results, indicating that dietary supplementation with 2% to 4% SCP-H16 enhances serum T-AOC and T-SOD activity, in addition to augmenting hepatic GSH-Px activity. These outcomes imply that the inclusion of SCP-H16 in the diet contributes to the enhancement of systemic antioxidant capacity in broilers.

The humoral immune response is also influenced by SCP-H16. This study demonstrates that substituting soybean meal with SCP-H16 in the diet leads to increased serum concentrations of IgG, IgA, and IgM, as well as elevated hepatic levels of IgG and IgA in broilers. Previous research has indicated that dietary SCP derived from *Clostridium autoethanogenum* enhances serum IgG levels in broilers [18], and that replacing soybean meal with rapeseed protein significantly increases serum IgA and IgM in piglets [19]. Additionally, prior studies have shown that SCP from *Chlorella vulgaris* and *Arthrospira platensis* enhances humoral immunity in poultry by increasing levels of IgA, IgM, and IgG [20]. These reports are consistent with the results of our current study. These findings suggest that SCP-H16 can enhance humoral immune parameters, as indicated by elevated serum and liver immunoglobulin levels.

Intestinal morphology is a crucial indicator for evaluating intestinal integrity and the efficiency of nutrient digestion and absorption, and it is significantly influenced by dietary composition. Prior research has demonstrated that the inclusion of 5% *Methylococcus*-derived SCP as a substitute for soybean meal substantially enhances villus height in the duodenum of broilers, while also positively affecting the morphology of the jejunum and ileum [21]. Additionally, the incorporation of rapeseed protein in place of soy protein concentrate or fish meal has been shown to significantly improve intestinal morphology in piglets, as indicated by increased villus height and an elevated villus height to crypt depth (VH/CD) ratio in the jejunum and ileum [19]. In the present study, the dietary inclusion of 2% and 4% SCP-H16 significantly increased the VH/CD ratio and reduced crypt depth in the duodenum and ileum of broilers. Notably, the 2% inclusion level also significantly increased duodenal villus height. These findings suggest that dietary supplementation with 2% to 4% SCP-H16 can enhance the intestinal architecture of broilers, and that inclusion of SCP-H16 at levels up to 8% does not adversely affect intestinal morphology.

The integrity of the intestinal epithelial barrier is predominantly maintained by tight junction proteins, whose functional levels have a direct impact on intestinal permeability. Previous research has demonstrated that substituting soybean meal with SCP derived from *Clostridium autoethanogenum* significantly upregulates the expression of intestinal tight junction protein genes, such as *occludin* and *ZO-1*, in grass carp [22]. Additionally, the mRNA expression levels of *ZO-1* and *occludin* in the intestinal tract of grass carp were markedly increased when soybean meal protein was replaced with Chlorella powder [23]. The present study indicates that dietary supplementation with SCP-H16, as a substitute for soybean meal, significantly enhances the mRNA expression of *occludin* and *ZO-1* in the duodenum and ileum, with the most pronounced effects at 2–4% inclusion. Critically, the spatial concordance between improved morphology and elevated tight junction transcripts in the duodenum and ileum implies that SCP-H16 may influence intestinal barrier function through both architectural remodeling and molecular consolidation, although functional improvement of the intestinal barrier cannot yet be established without direct measurements of intestinal permeability or tight-junction protein abundance.

A significant finding of this study is the non-linear dose–response relationship observed. The most consistent beneficial effects on feed efficiency, antioxidant status, immunity, and intestinal morphology were noted at SCP-H16 inclusion levels of 2–4%, whereas higher inclusion levels at 6–8% exhibited reduced or absent improvements. Several mechanisms may account for this pattern. Firstly, although diets were formulated to be isonitrogenous, potential imbalances in amino acid profiles or differences in amino acid bioavailability between SCP-H16 and soybean meal may become limiting at higher inclusion rates. Secondly, bacterial cell wall components, such as lipopolysaccharides (LPS), or other anti-nutritional factors present in *R. eutropha* biomass, may exert detrimental effects when SCP-H16 exceeds a certain threshold. Thirdly, the birds’ digestive and metabolic capacity to process bacterial protein may become saturated at elevated levels. Future research assessing nutrient digestibility, microbiota composition, and inflammatory markers across the full range of inclusion levels would be instrumental in elucidating these mechanisms.

## 5. Conclusions

In summary, this study demonstrates that SCP-H16 can be included at up to 8% of the diet as a partial replacement for soybean meal without compromising final body weight or meat quality, with 2–4% inclusion conferring additional benefits including increased feed conversion efficiency, reduced serum cholesterol, enhanced antioxidant capacity, elevated humoral immunity, and improved intestinal morphology and barrier-related gene expression. Based on these findings, a dietary inclusion level of 2% to 4% SCP-H16 appears promising as a partial soybean meal substitute. However, further studies incorporating economic analysis, amino acid digestibility assessments, and commercial-scale validation are warranted before definitive practical recommendation. Overall, this study suggests that SCP-H16 is a promising alternative protein source for the partial substitution of soybean meal in broiler diets.

## Figures and Tables

**Table 1:** Composition and nutrient levels of diets for 1–21 d (air-dry basis).

Items	Control	SCP-H16			
		2%	4%	6%	8%
Ingredient					
Corn	47.95	49.48	51.01	52.54	54.07
Soybean meal, 43% CP	37.29	33.94	30.59	27.23	23.88
SCP-H16		2.00	4.00	6.00	8.00
Corn gluten meal, 60% CP	5.00	5.00	5.00	5.00	5.00
Soybean oil	4.72	4.46	4.20	3.95	3.69
CaHPO_4_	1.44	1.50	1.55	1.61	1.67
Limestone	1.08	1.07	1.06	1.06	1.05
*L*-Lysine	0.64	0.67	0.69	0.71	0.74
Sodium chloride	0.33	0.33	0.34	0.34	0.34
*DL*-Methionine	0.30	0.31	0.31	0.32	0.33
Sodium bicarbonate	0.15	0.15	0.15	0.15	0.15
*L*-Threonine	0.09	0.09	0.08	0.08	0.07
*L*-Tryptophan		0.01	0.01	0.02	0.02
Premix ^1^	1.00	1.00	1.00	1.00	1.00
Total	100	100	100	100	100
Nutrient levels ^2^					
Metabolizable energy, MJ/kg	12.34	12.34	12.34	12.34	12.34
Crude protein, %	23.68	23.27	23.47	23.80	23.31
Calcium, %	0.94	0.98	1.05	1.10	1.00
Total Phosphorus, %	0.68	0.62	0.61	0.64	0.67
Lysine, %	1.5	1.5	1.5	1.5	1.5
Methionine, %	0.67	0.67	0.67	0.67	0.67
Threonine, %	0.95	0.95	0.95	0.95	0.95
Tryptophan, %	0.25	0.25	0.25	0.25	0.25

^1^ The premix provided the following per kilogram diet: vitamin A, 32,000 IU; vitamin D3, 10,000 IU; vitamin E, 100 mg; vitamin K3, 15 mg; vitamin B1, 10 mg; vitamin B2, 30 mg; vitamin B6, 20 mg; vitamin B12, 100 μg; pantothenic acid, 60 mg; nicotinamide, 200 mg; folacin, 5 mg; biotin, 0.5 mg; choline, 700 mg; Zn, 90 mg; Fe, 110 mg; Cu, 20 mg; Mn, 100 mg; I, 0.5 mg; phytase, 0.1 g. ^2^ Crude protein, calcium, and phosphorus are measured values; all others are calculated values using data provided by the China Feed Database (2020) [13].

**Table 2:** Composition and nutrient levels of diets for 22–42 d (air-dry basis).

Items	Control	SCP-H16			
		2%	4%	6%	8%
Ingredient					
Corn	49.24	50.77	52.30	53.83	55.36
Soybean meal, 43% CP	32.67	29.32	25.97	22.61	19.26
SCP-H16		2.00	4.00	6.00	8.00
Corn gluten meal, 60% CP	5.00	5.00	5.00	5.00	5.00
Soybean oil	8.91	8.66	8.40	8.15	7.89
CaHPO_4_	0.81	0.87	0.92	0.98	1.03
Limestone	1.08	1.07	1.06	1.05	1.04
*L*-Lysine	0.49	0.52	0.54	0.57	0.59
Sodium chloride	0.26	0.26	0.26	0.26	0.26
*DL*-Methionine	0.30	0.31	0.32	0.32	0.33
Sodium bicarbonate	0.15	0.15	0.15	0.15	0.15
*L*-Threonine	0.08	0.08	0.08	0.07	0.07
*L*-Tryptophan		0.01	0.01	0.02	0.02
Premix ^1^	1.00	1.00	1.00	1.00	1.00
Total	100	100	100	100	100
Nutrient levels ^2^					
Metabolizable energy, MJ/kg	13.60	13.60	13.60	13.60	13.60
Crude protein, %	21.44	21.39	21.58	21.77	21.69
Calcium, %	0.75	0.73	0.74	0.78	0.70
Total Phosphorus, %	0.45	0.46	0.40	0.50	0.48
Lysine, %	1.30	1.30	1.30	1.30	1.30
Methionine, %	0.65	0.65	0.65	0.65	0.65
Threonine, %	0.87	0.87	0.87	0.87	0.87
Tryptophan, %	0.21	0.21	0.21	0.21	0.21

^1^ The premix provided the following per kilogram diet: vitamin A, 10,000 IU; vitamin D3, 3000 IU; vitamin E, 50 IU; vitamin K3, 3.5 mg; vitamin B1, 2 mg; vitamin B6, 5 mg; vitamin B12, 6 μg; pantothenic acid, 20 mg; nicotinamide, 60 mg; folacin, 8 mg; biotin, 0.6 mg; choline, 600 mg; Zn, 80 mg; Fe, 100 mg; Cu, 15 mg; Mn, 80 mg; I, 0.5 mg; phytase, 0.1 g. ^2^ Crude protein, calcium, and phosphorus are measured values; all others are calculated values using data provided by the China Feed Database (2020) [13].

**Table 3:** Effects of SCP-H16 on the growth performance of broilers.

Items	Control	SCP-H16 Inclusion Level				SEM	*p*-Value
		2%	4%	6%	8%		
BW, g							
21 d	814.72 ^a^	731.05 ^b^	740.74 ^b^	700.00 ^b^	687.26 ^b^	10.258	<0.001
42 d	2385.30	2365.47	2390.28	2304.36	2282.46	15.091	0.062
1~21 d							
ADG, g	36.65 ^a^	32.67 ^b^	33.13 ^b^	31.19 ^b^	30.58 ^b^	0.488	<0.001
ADFI, g	44.60 ^a^	41.04 ^ab^	40.88 ^ab^	39.44 ^b^	39.19 ^b^	0.612	0.027
FCR	1.22	1.26	1.23	1.27	1.28	0.009	0.130
22~42 d							
ADG, g	74.79	77.83	78.55	75.93	75.96	0.607	0.282
ADFI, g	134.58 ^a^	126.03 ^b^	129.91 ^ab^	126.24 ^b^	124.85 ^b^	0.967	0.003
FCR	1.80 ^a^	1.62 ^b^	1.65 ^b^	1.66 ^b^	1.64 ^b^	0.014	<0.001
1~42 d							
ADG, g	55.72	55.25	55.84	53.80	53.27	0.359	0.062
ADFI, g	89.59 ^a^	83.54 ^b^	85.40 ^b^	82.83 ^b^	82.02 ^b^	0.648	<0.001
FCR	1.61 ^a^	1.51 ^b^	1.53 ^b^	1.55 ^b^	1.54 ^b^	0.008	<0.001

^a, b^ Means in the same row followed by different superscript letters differ significantly (*p* < 0.05). Data are presented with the mean ± pooled SEM (*n* = 6). BW: bodyweight; ADG: average daily gain; ADFI: average daily feed intake; FCR: feed conversion ratio.

**Table 4:** Effects of SCP-H16 on the breast meat quality of broilers.

Items	Control	SCP-H16 Inclusion Level				SEM	*p*-Value
		2%	4%	6%	8%		
pH _45min_	6.57	6.69	6.46	6.62	6.44	0.033	0.069
L* _45min_	55.03	57.60	54.20	56.72	58.71	0.632	0.138
a* _45min_	5.19	5.94	6.54	6.07	5.12	0.223	0.230
b* _45min_	11.29	11.53	11.36	12.87	11.27	0.281	0.326
pH _24h_	5.70	5.61	5.85	5.71	5.86	0.054	0.547
L* _24h_	60.05	63.61	61.39	62.24	60.96	0.530	0.275
a* _24h_	6.63	6.58	7.38	7.29	6.60	0.260	0.777
b* _24h_	12.78	12.96	14.55	14.37	12.79	0.641	0.579
Drip loss (%)	1.65	1.74	1.72	1.66	1.96	0.111	0.917
Cooking loss (%)	25.51	26.45	25.02	27.60	26.70	0.476	0.483

Data are presented with the mean ± pooled SEM (*n* = 6). L*, lightness; a*, redness; b*, yellowness.

**Table 5:** Effects of SCP-H16 on the serum biochemical indices of broilers.

Items	Control	SCP-H16 Inclusion Level				SEM	*p*-Value
		2%	4%	6%	8%		
ALT (U/L)	9.35	9.05	8.53	9.13	8.93	0.304	0.949
AST (U/L)	323.75	321.62	315.10	276.51	273.74	9.799	0.278
ALP (U/L)	1461.67	1422.50	1448.33	1225.83	1431.67	57.709	0.712
T-CHO (mmol/L)	2.32 ^a^	1.62 ^b^	1.71 ^b^	1.89 ^ab^	1.88 ^ab^	0.068	0.005
UA (μmol/L)	276.37	274.07	251.37	259.40	254.07	3.548	0.064
CREA (μmol/L)	20.33	17.17	14.83	17.67	16.33	0.785	0.262
TBIL (μmol/L)	3.90	3.60	3.50	3.55	3.22	0.113	0.464
TP (g/L)	24.22	25.82	26.18	24.87	25.07	0.654	0.903
ALB (g/L)	11.20	12.73	13.77	12.80	13.70	0.436	0.354
GLO (g/L)	13.60	12.03	12.53	12.10	11.90	0.443	0.768
GLU (mmol/L)	8.83	8.64	8.65	8.77	8.31	0.155	0.872

^a, b^ Means in the same row followed by different superscript letters differ significantly (*p* < 0.05). Data are presented with the mean ± pooled SEM (*n* = 6). ALT, alanine aminotransferase; AST, aspartate aminotransferase; ALP, alkaline phosphatase; TP, total protein; ALB, albumin; GLO, globulin; T-CHO, total cholesterol; GLU, glucose; TBIL, total bilirubin; UA, uric acid; CREA, creatinine.

**Table 6:** Effects of SCP-H16 on the antioxidant capacity of serum and liver in broilers.

Items	Control	SCP-H16 Inclusion Level				SEM	*p*-Value
		2%	4%	6%	8%		
Serum							
T-SOD (U/mL)	61.71 ^b^	70.72 ^a^	71.05 ^a^	69.12 ^a^	66.88 ^ab^	0.908	0.002
T-AOC (mmol/L)	0.45 ^b^	0.51 ^a^	0.50 ^a^	0.47 ^ab^	0.44 ^b^	0.008	0.003
MDA (nmol/mL)	4.11	3.02	3.18	4.07	4.20	0.207	0.196
GSH-Px (U/mL)	787.06	819.88	807.00	803.44	769.09	6.255	0.083
Liver							
T-SOD (U/mgprot)	212.88	232.09	241.65	235.31	228.92	8.216	0.869
T-AOC (mmol/gprot)	0.05	0.06	0.05	0.05	0.05	0.001	0.101
MDA (nmol/mgprot)	2.11	1.82	1.74	1.81	1.92	0.046	0.090
GSH-Px (U/mgprot)	44.66 ^b^	59.72 ^a^	54.40 ^ab^	50.22 ^ab^	46.38 ^b^	1.623	0.010

^a, b^ Means in the same row with different superscript letters differ significantly (*p* < 0.05). Data are presented with the mean ± pooled SEM (*n* = 6). T-SOD, total superoxide dismutase; T-AOC, total antioxidant capacity; MDA, malondialdehyde; GSH-Px, glutathione peroxidase.

**Table 7:** Effects of SCP-H16 on immunoglobulin levels in serum and liver of broilers.

Items	Control	SCP-H16 Inclusion Level				SEM	*p*-Value
		2%	4%	6%	8%		
Serum							
IgG (μg/mL)	368.80 ^b^	499.32 ^a^	481.59 ^a^	485.58 ^a^	404.98 ^b^	11.689	<0.001
IgA (μg/mL)	36.95 ^b^	46.46 ^a^	48.62 ^a^	50.56 ^a^	51.86 ^a^	1.330	<0.001
IgM (μg/mL)	91.09 ^c^	126.01 ^a^	114.71 ^a^	110.69 ^ab^	93.40 ^bc^	4.626	<0.001
Liver							
IgG (μg/mgprot)	123.82 ^b^	167.00 ^a^	162.01 ^a^	190.17 ^a^	184.91 ^a^	5.776	<0.001
IgA (μg/mgprot)	14.12 ^b^	20.15 ^a^	20.44 ^a^	19.66 ^a^	21.91 ^a^	0.696	<0.001
IgM (μg/mgprot)	39.61	40.13	40.07	42.93	41.16	0.771	0.676

^a, b, c^ Means in the same row different superscript letters differ significantly (*p* < 0.05). Data are presented with the mean ± pooled SEM (*n* = 6). IgA, immunoglobulin A; IgG, immunoglobulin G; IgM, immunoglobulin M.

**Table 8:** Effects of SCP-H16 on the intestinal morphology of broilers.

Items	Control	SCP-H16 Inclusion Level				SEM	*p*-Value
		2%	4%	6%	8%		
Duodenum							
VH, μm	1880.43 ^b^	2097.08 ^a^	1992.38 ^ab^	1885.36 ^b^	1876.86 ^b^	20.018	<0.001
CD, μm	261.64 ^a^	225.55 ^b^	221.69 ^b^	246.03 ^ab^	256.17 ^a^	4.018	<0.001
VH/CD	7.21 ^b^	9.32 ^a^	9.01 ^a^	7.70 ^b^	7.36 ^b^	0.188	<0.001
Jejunum							
VH, μm	1460.69	1484.09	1513.89	1452.87	1427.48	11.798	0.185
CD, μm	262.13	245.43	234.20	250.39	254.76	4.761	0.445
VH/CD	5.61	6.08	6.48	5.86	5.69	0.112	0.085
Ileum							
VH, μm	1278.02	1293.90	1340.21	1279.81	1246.76	13.825	0.315
CD, μm	236.93 ^a^	194.42 ^b^	188.10 ^b^	205.16 ^ab^	231.41 ^a^	4.934	<0.001
VH/CD	5.42 ^b^	6.72 ^a^	7.14 ^a^	6.28 ^ab^	5.41 ^b^	0.157	<0.001

^a, b^ Means in the same row with different superscript letters differ significantly (*p* < 0.05). Data are presented with the mean ± pooled SEM (*n* = 6). VH, villus height; CD, crypt depth; VH/CD, villus height/crypt depth.

**Table 9:** Effects of SCP-H16 on the relative mRNA expression of tight junction-related genes of broilers.

Items	Control	SCP-H16 Inclusion Level				SEM	*p*-Value
		2%	4%	6%	8%		
Duodenum							
*Occludin*	0.83 ^b^	1.12 ^a^	0.92 ^b^	0.80 ^b^	0.78 ^b^	0.030	<0.001
*Claudin-1*	1.06	1.16	1.21	1.17	1.07	0.026	0.300
*ZO-1*	0.94 ^b^	1.16 ^a^	1.02 ^ab^	0.85 ^b^	0.83 ^b^	0.031	<0.001
Jejunum							
*Occludin*	0.78	0.81	0.79	0.74	0.65	0.028	0.410
*Claudin-1*	0.64	0.73	0.76	0.68	0.68	0.021	0.404
*ZO-1*	0.69 ^ab^	0.84 ^a^	0.72 ^ab^	0.68 ^ab^	0.60 ^b^	0.025	0.038
Ileum							
*Occludin*	0.62 ^b^	0.82 ^a^	0.78 ^a^	0.64 ^b^	0.61 ^b^	0.021	<0.001
*Claudin-1*	0.85	0.96	1.00	0.91	0.88	0.020	0.119
*ZO-1*	0.81 ^b^	1.01 ^a^	0.92 ^ab^	0.84 ^ab^	0.79 ^b^	0.025	0.013

^a, b^ Means in the same row followed by different superscript letters differ significantly (*p* < 0.05). Data are presented with the mean ± pooled SEM (*n* = 6). *ZO-1*, zonula occludens-1.

## Data Availability

All data in this study are available from the authors upon reasonable request.

## Supplementary Materials

The following supporting information can be downloaded at: https://www.mdpi.com/article/10.3390/ani16172769/s1, Table S1: Chemical composition of the *Ralstonia eutropha* H16 SCP; Table S2: Primer sequences used in this experiment.
