# Supplementary material for: Effects of Single-Cell Protein Derived from Ralstonia eutropha on Growth Performance, Meat Quality, Antioxidant Capacity, and Intestinal Health in Broilers
Source: Animals (Basel). 2026 Sep 3;16(17):2769. doi: 10.3390/ani16172769 (PMC13564502; doi:10.3390/ani16172769)
Supplement: Supplementary file 1 [file animals-16-02769-s001.zip › animals-4544220-supplementary.pdf]

## Supplementary Materials

**Table S1.** Chemical composition of the *Ralstonia eutropha* H16 SCP (%, air-dry basis)

| Items | Content |
|---|---|
| Routine chemical components | |
| Metabolizable energy, MJ/kg | 11.5 |
| CP, % | 69.1 |
| EE, % | 0.6 |
| Ash, % | 3.8 |
| Amino acids | |
| Asp, % | 5.33 |
| Thr, % | 2.78 |
| Ser, % | 2.15 |
| Glu, % | 7.34 |
| Gly, % | 4.14 |
| Ala, % | 5.95 |
| Val, % | 4.13 |
| Met, % | 1.26 |
| Ile, % | 2.80 |
| Leu, % | 5.11 |
| Tyr, % | 1.96 |
| Phe, % | 2.86 |
| His, % | 1.21 |
| Lys, % | 3.65 |
| Arg, % | 4.45 |
| Pro, % | 2.82 |
| TAA, % | 57.95 |

CP, crude protein; EE, ether extract; Asp, aspartic acid; Thr, threonine; Ser, serine; Glu, glutamic acid; Gly, glycine; Ala, alanine; Val, valine; Met, methionine; Ile, isoleucine; Leu, leucine; Tyr, tyrosine; Phe, phenylalanine; His, histidine; Arg, arginine; Lys, lysine; Pro, proline. TAA, total amino acids.

**Table S2**. Primer sequences used in this experiment

| Gene | Primers sequence (5′→3′) | Accession no. |
|---|---|---|
| *GAPDH* | F: AGCCATTCCTCCACCTTTGAT | NM_204305.2 |
| | R: AGTCCACAACACGGTTGCTGTAT | |
| *Occludin* | F: GATGGACAGCATCAACGACC | NM_205128.1 |
| | R: CTTGCTTTGGTAGTCTGGGC | |
| *Claudin-1* | F: ACACCCGTTAACACCAGATTT | NM_001013611.2 |
| | R: GCATTTTTGGGGTAGCCTCG | |
| *ZO-1* | F: GCCTACTGCTGCTCCTTACAACTC | XM_040680630.1 |
| | R: GCTGGATCTATATGCGGCGGTAAG | |

*GAPDH*, glyceraldehyde-3-phosphate dehydrogenase; *ZO-1*, zonula occludens-1
